# Evolutionary adaptation of trees and modelled future larch forest extent in Siberia

**DOI:** 10.1016/j.ecolmodel.2023.110278

**Published:** 2023-04

**Authors:** Josias Gloy, Ulrike Herzschuh, Stefan Kruse

**Affiliations:** a Polar Terrestrial Environmental Systems Research Group, Alfred Wegener Institute, Helmholtz Centre for Polar and Marine Research, 14473 Potsdam, Germany; b Institute of Biology and Biochemistry, University of Potsdam, 14476 Potsdam, Germany; c Institute of Earth and Environmental Science, University of Potsdam, 14476 Potsdam, Germany

**Keywords:** Adaptive trait, Conifer, Individual-based simulation, *Larix*, Simulation, Spatially explicit, Trait variation, Vegetation model

## Abstract

With changing climate, the boreal forest could potentially migrate north and become threatened by droughts in the south. However, whether larches, the dominant tree species in eastern Siberia, can adapt to novel situations is largely unknown but is crucial for predicting future population dynamics. Exploring variable traits and trait adaptation through inheritance in an individual-based model can improve our understanding and help future projections.

We updated the individual-based spatially explicit vegetation model LAVESI (*Larix* Vegetation Simulator), used for forest predictions in Eastern Siberia, with trait value variation and incorporated inheritance of parental values to their offspring. Forcing the model with both past and future climate projections, we simulated two areas – the expanding northern treeline and a southerly area experiencing drought. While the specific trait of ‘seed weight’ regulates migration, the abstract ‘drought resistance’ protects stands.

We show that trait variation with inheritance leads to an increase in migration rate (∼ 3% area increase until 2100). The drought resistance simulations show that, under increasing stress, including adaptive traits leads to larger surviving populations (17% of threatened under RCP 4.5 (Representative Concentration Pathway)). We show that with the increase expected under the RCP 8.5 scenario vast areas (80% of the extrapolated area) of larch forest are threatened and could disappear due to drought as adaptation plays only a minor role under strong warming.

We conclude that variable traits facilitate the availability of variants under environmental changes. Inheritance allows populations to adapt to environments and promote successful traits, which leads to populations that can spread faster and be more resilient, provided the changes are not too drastic in both time and magnitude. We show that trait variation and inheritance contribute to more accurate models that can improve our understanding of responses of boreal forests to global change.

## Highlights

• The implementation of variation and adaptation of traits in a vegetation model changes the results.
• The northern treeline of Siberia could be advancing faster than previously predicted.
• The larches of central Siberia cannot withstand future drought even if they adapted.

## 1 Introduction

With the expected climate warming, the boreal forest of eastern Siberia will potentially undergo changes in the near future (Bonan 2008). The warming of the climate may lead to a shift in range towards the north (Mamet et al., 2019) and an overall reduction in area due to drought diebacks in the south (Villén-Peréz et al., 2020). As one of the largest terrestrial ecosystems, changes in, or especially a collapse of, the boreal forest could have far-reaching effects both with regard to the release of stored carbon (Schaphoff et al. 2016) and in the change in albedo (Bonan 2008). To predict future boreal forest response, simulations should be as accurate as possible (Aubin et al., 2016). While acknowledging that models need to make simplifications, for example assuming uniform trees, one area that could be improved is trait variability as this can have large impacts on the outcome of predictions (Verheijen et al., 2015). To allow for variation over time and to assign more realistic trait values, adaptation of traits would further improve the models. This could serve to limit the overestimation of trait variation and allow for plasticity between generations.

Northern leading-edge range expansion: one of the results of a warming climate is the expansion of tree-species into areas previously too cold for survival (Mamet et al., 2019), as warmer climate leads to better growth (Zhang et al., 2019). The northern forest line has historically always shifted with changes in climate and is expected to continue to do so (McDonald et al., 2008). While estimates of the potential range of species could be done using climate constraints, this would neglect other factors. Since trees are immobile and seed dispersal is a limiting factor in range expansions of forest populations, a fast-advancing potential range can lead to a time lag effect where the actual treeline is behind its potential position (Lloyd 2005). Due to this, a simple model that lacks spatial dispersion cannot predict future distributions. Hence, vegetation modelling that can address these questions is needed. A further factor is the variation in seed weight (e.g. Wieczorek et al., 2017, Barchenkov 2011), as different seeds can be transported different distances by the wind due to their weight, with heavier seeds having a higher descent speed (Debain et al., 2003; Greene and Johnson 1993; Augspurger and Franson 1987).

Southern rear-edge drought-dieback: projected increases of droughts are assumed to reduce the forested area and give way to steppe vegetation (Mamet et al., 2019). While the increase of temperature is expected to coincide with an increase in precipitation, current predictions indicate that this will be of a smaller magnitude and thus still result in a more arid climate (Harris et al., 2020). These data also show that the effect is not limited to the southern edge of the distribution area, but also applies to other areas, such as central Yakutia. Generally, trees are locally able to adapt to drought conditions at the cost of decreased growth (McDowell et al., 2008; Read and Stokes 2006), as found for populations of L. *gmelinii* (Kagawa et al., 2006) and other species of larch in Siberia (Zhirnova et al., 2020)*.* However, it is not known how well populations would be able to adapt to predicted environmental changes and where populations could survive. This has a strong impact on carbon storage and overall global warming; therefore, it is important to be able to include adaptation into models.

Both the expansion north (Chapin and Starfield 1997; Kruse et al., 2016, 2022) and the drought in the south (Brazhnik and Shugart 2015) have been studied previously using models to predict future development. One of these models is the individual-based spatially explicit model LAVESI (*Larix* Vegetation Simulator) that was development for simulating the migration north of *Larix gmelinii* (Kruse et al., 2016) and has since been expanded to include further boreal forest species among other (Kruse et al., 2022). For the north it is estimated that the larches will replace the tundra (Kruse and Herzschuh 2022), but that the time lag created by the migration of the trees could lead to the formation of grassland (Chapin and Starfield 1997). The droughts are expected to reduce the forested area in the south (Brazhnik and Shugart 2015). The models covering this region are rather few though and do not include adaptation of traits.

Trait variation is a parameter different types of vegetation modellers are striving to include: Dynamic Global Vegetation Models (DGVMs) are designed and tuned for global simulations and are historically mainly concerned with plant functional types (Maréchaux et al., 2021). Due to limitation in the computational resources, they often do not include dispersal, like Scheiter and Higgins (2009). There are models like aDGVM2 (Scheiter et al., 2013) that include trait variation of plant functional types and allow for adaptation over time. This process is, however, limited to a community level for the trait pool and thus does not include scale seed distribution. Even in models using individual trees with trait variation there are still restrictions due to their abstract implementation of vegetation (Sakeschewski et al., 2015; Scheiter and Higgins 2009). Another option is to work with smaller scaled individual-based models (IBMs) that simulate all individual trees and all life-history stages in a given area but are therefore limited in the size of the area they can simulate. Such models allow for the inclusion of other small-scale interactions such as explicit dispersal for both seeds and pollination. These can then be used to simulate adaptation of traits across a landscape in response to their environment. One model that does include traits variation but not adaptation is GRASSMIND, which, as the name implies, is focused on grasses and, as such, at a fine scale (Schmid et al., 2021). As pointed out by Romero-Mujali et al. (2019) there are still only a few models that include adaptation and most of these do not simulate actual species but rather abstract model species.

Here, we adapt the model LAVESI to evaluate the impact of trait variation and inheritance on key variables of larch forests of eastern Eurasia: survival in the south by incorporating changes in drought resistance and migration in the north by considering differences in seed weights. We aim to understand the dynamics of boreal larch forest in a warming eastern Eurasia, and formulated the following specific research questions:

- Will the migration and survival of larches be positively affected by our implementation of trait variation and adaptation in the model?
- What is the effect of adaptive traits on larch forest distribution in Siberia under a warming climate?

## 2 Methods

### 2.1 General model description

We selected for our study the individual-based spatially explicit model LAVESI that was developed to simulate population dynamics of widespread *Larix gmelinii* in north-eastern Siberia (Kruse et al., 2016) and developed for treeline migration with a parametrisation for the Taymyr region (Kruse et al., 2016; Wieczorek et al., 2017). It simulates the life cycle of all trees in a given area for each year, starting from the seed stage. Hence, the model already includes the study species and was recently further equipped with new functions, which lay the groundwork for this study. Besides seed dispersal, wind-dependent and spatially explicit pollination was introduced (Kruse et al., 2018).

In short, the model simulates every year by going through the following sub-routines, established in previous publications of the model (Kruse et al., 2016; Kruse et al., 2018):

*Initialisation:* The environmental information (elevation, slope, terrain water index) of the simulated area is read in and filled in the model's structures. The weather provided in monthly temperature and precipitation values is read for a given number of years. Each simulation run starts with an empty area and seeds are introduced to the area during a spin-up period at the start of the model.

*Environment is updated*: As density map is calculated for the area in which the density influence is recorded for the trees. The competition between trees is calculated based on the basal diameter in a given area of the density map (Formula 1). The active-layer depth is estimated based on the number of days exceeding 0 °C.

(1) $$density\;influence\left(x-coord,y-coord\right)=\;\frac{diameter_{basal}}{\sqrt{x-coord^{2}+y-coord^{2}}+1}$$

*Growth*: The maximal growth is calculated based on an average of ten years’ climate data. The basal growth (Formula 2) of one individual for the year is then derived from this by including the density index of the tree. Based on the diameter, the tree height is estimated (Formula 3).

(2) $$Growth_{max,diameter}\;=\left(precipitation*Growth_{standard,diameter}*\left(\frac{1}{f_{AATNDD}}*AATI+\left(1-\frac{1}{f_{AATNDD}}\right)*net\;degree\;days\right)\right)*\left(1-TDEI\right)$$

(3) $$height=\left\{ \begin{array}{cc} 44.43163*diameter_{basal} & ,\;\;height<1.3\;m\\ {\left(7.02*\sqrt{diameter_{breast}}\right)}^{2}+130 & \;,\;\;height\geq 1.3\;m \end{array}\right.$$

*Seed dispersal*: Seeds that are still within cones are dispersed, the direction and distance are randomly determined influenced by wind data (Formula 4). When seeds leave the extent of the transect to either the east or west they are reintroduced from the opposite site, to simulate a larger forest and avoid the loss of many seeds.

(4) $$\text{distance}=\sqrt{2{\left(\text{release}\;\text{height}\cdot \frac{\text{wind}\;\text{speed}}{\text{fall}\;\text{speed}}\right)}^{2}\;\left(-\text{log}\left(\text{rand}\right)\right)+\frac{1}{2}\text{distanceratio}\cdot \;\text{ran}d^{-1.5}}$$

*Seed production*: Once a tree has reached the height of maturation, based on a pre-generated distribution randomly assigned, it produces seeds. The amount produced in each year is based on the height of the tree, competition, and the weather (Formula 5).

(5) $$\text{seeds}\;\text{produced}=\left\lfloor \text{facto}r_{S}\;*\text{Diamete}r_{\text{basal}}*\left(1.0-{\left(\frac{\text{height}}{50\;\;m}\right)}^{-1.0}\right)\right\rfloor$$

*Establishment*: Seeds that are on the ground germinate based on weather conditions (Formula 6).

(6) $$probability\;to\;germinate\;\left(year\right)=background\;germination\;rate+\left(weather\;quality\;factor*\frac{G_{rowthmax,basal}\left(year\right)}{Growth_{standard,basal}}\right)$$

*Mortality*: The probability of death is calculated for each tree and, based on that, it is semi-randomly determined whether the tree dies and is removed from the simulation. The calculation is based on long-term weather values, the calculated drought strength, competition of surrounding trees, the age and size of the tree, and a base mortality rate. For each of these a mortality value is calculated, these are then summed up and compared to a randomly generated number. If the sum of death probabilities is larger the tree dies. The death of seeds is determined at this step as well, although the mortality rate for these is fixed.

*Ageing*: The last step is an increase in the age of both the seeds and the trees. Since every year is simulated the age is advanced by once each cycle. The seeds are removed once they have reached a certain (3 years) age limit.

### 2.2 New model development

The new features of the model are variation and inheritance of traits. In a previous version of the model LAVESI (Kruse et al., 2016), almost all traits were the same for every individual (only maturation height being randomly assigned) of the same species, which is referred to as the uniform type throughout this article. In this work, we created variation and adaptation for the seed weight and the drought resistance. Both use the same following principles in their trait value determination.

Trait variation gives every newly produced seed a random value from a uniform distribution with upper and lower limits. Therefore, every seed that is either introduced or created by a tree during the simulation has the same chance of having any trait value from the distribution. This allows for variability in the traits present in the model permitting plastic responses but no adaptation while not being computationally much more intensive.

**Fig. 1:**
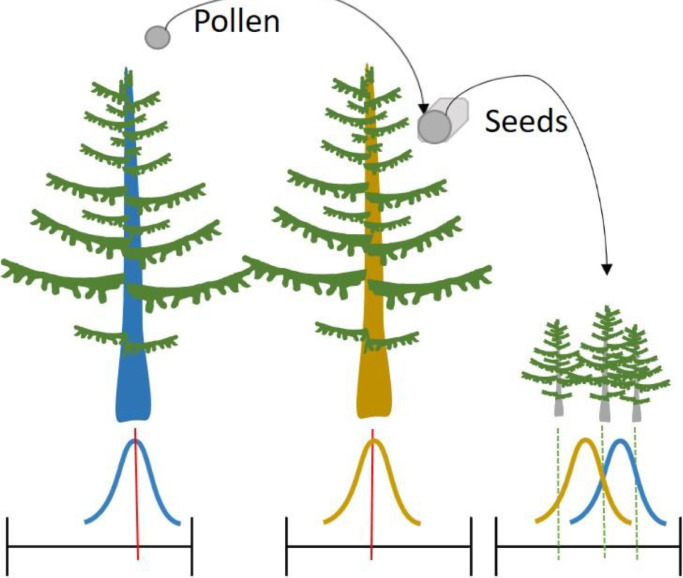
Scheme showing the inheritance system. The upper part shows a schematic path from the pollen to the seed-producing tree and from the seeds to the offspring. Red vertical lines in the lower part show the trait values of individuals within a range of possible trait values represented by the black bars along the x axis. The specific trait values are examples and could be anywhere within the range. The blue curve shows the distribution of likely trait values, around the individuals own trait value, for the offspring based on the pollen and the orange based on the seed-producing tree. The right shows the offspring uses a mixed distribution resulting from these two to determine their trait values. The green dotted lines for the offspring show three possible trait values as examples that could be created by these two parent trees.

The inheritance (adaptive traits) system (Fig. 1), on the other hand, calculates a new seed value for every new seed production that is a function of the trait value of the mother tree and the pollen source (Geber and Griffen 2003). To calculate the likely pollen source, it was necessary to create a level of abstraction as calling every tree for every seed production proved too computationally intensive (Kruse et al., 2018). For this reason, a pollen grid was introduced with grid cells of, in this case, 100 m². The trait information of the pollen-producing trees is averaged per cell and these mean grid-cell values used as the trait values for that pollen source. The new seed's trait value is calculated by either using a mixture distribution, created by combining the two parental normal distributions, or by a normal distribution around a weighted average of the pollen source and the seed-producing tree. For the normal distributions the Box-Muller transform is used (Box and Muller, 1958). The mixed distribution is achieved by creating normal distributions for both parents and randomly selecting one or the other. For the northern tree expansion both methods are used and two different weights are applied to the second method, with the weight applied being equal in one case and 75% for the seed-producing tree in the other case, to assess the different options. Since all three methods produce comparative results (not shown) only the mixed distribution is used for the drought experiment.

## 3 Experiment for northward treeline migration

### 3.1 Model development for the migration

The model is parametrised for the Taymyr region in Siberia and could be used directly for the northward migration simulations (Kruse et al., 2016; Kruse et al., 2019). Taymyr is at the northern edge of the distribution area of L. *gmelinii* where the species is expanding farther north as the climate warms (McDonald et al., 2008).

For the northward migration, the trait of interest is seed weight, not in units of grams but rather as relative weight to each other. This trait influences three factors – dispersal distance, the number of seeds a tree produces, and the germination rate – with the first two being negatively correlated to weight and the last one positively. The actual weight of a seed is not determined by the trait information the seeds carry but rather by the tree producing the seeds. When seeds are introduced at the start of the simulation or when a random variation is assigned, a seed is randomly given a normally distributed selected weight value, with the average being one and the upper and lower boundaries being 5/3 and 1/3. These trait value limits for seed weight also hold true for seeds created using adaptation.

The three impacts in the model of the seed weight are all based on the relative seed weight. The number of seeds produced by a tree is divided by the squared weight, determined by the trees seed weight value, of those seeds (seed number/ (seed weight^2^)). The germination of seeds is multiplied by 0.12 and the seed weight from which 1 is subtracted. This subtraction leads to a decrease with seeds below 1 and an increase when seeds with a weight above 1 are used. When the seed weight is exactly average there is neither a positive nor negative effect (germination*0.12*(seed weight −1)). The extent of the effect was based on a study by Gorian et al. (2007). The distance is divided by the square root or the seed weight ($distance/\sqrt{seedweight}$).

### 3.2 Simulations for the migration

Simulation runs are performed using a transect representing a slice of the northern forest line with a size of 10 km x 0.2 km and seeds being reintroduced when leaving to the eastern or western side to simulate the continuation of the forest in either direction. The simulations are forced for 2000 years using randomised weather taken from the period 1901–2018 from CRU TS4.0 (Harris et al., 2020) for the location (98 °E 70.66 °N) with the temperature for the site being lowered by 0.5 °C. This location is close to the treeline and model test runs indicated that it has a climate that is barely above the required minimum for tree growth. The decrease in temperature was introduced to bring the temperature to the very edge: if the temperature was decreased further no growth occurred. In the first 50 years, 2000 seeds per year were introduced from the south with a negative exponential dispersal function and given trait values according to one of the three scenarios: uniform, variation, and inheritance. For model spin up, the population was restricted during the first 500 years to the southern 1 km x 0.2 km to allow a population to establish. Afterwards the entire area becomes uniformly inhabitable. Each of the simulation scenarios was repeated 30 times to counteract variation.

To compare migration rates under the scenarios a quasi-binomial model was established, using the percentage of the area south of the forest line along the 10 km long transect over time. The forest line is defined as the northernmost position at which forest density does not fall below a threshold of one tree per hectare where a tree has to be >1.3 m tall. The statistical analysis was performed using the statistical tool R (R Core Team, 2020).

The mapping of the effect of seed-weight adaptation on the broader scale was conducted using the results of a large-scale simulation based on a uniform trait model by Kruse and Herzschuh (2022) as our baseline. In detail, the simulated treeline position through time over 2000–3000 CE (Common Era) stemming from the transect study covering the region between the current treeline and the Arctic Ocean was modified using the binomial curves obtained in this study for the adaptation. The two variants are then shown on a map of the area. This approach was chosen because simulation of a transect of the required length with the higher temperatures was unmanageable in the currently unoptimised version, as the required computational power was too large to cope with the increase of trees.

## 4 Experiment for southern tree die-back

### 4.1 Model development for the drought

For simulations of the southern region prone to increasing drought in a warming climate, the area of central Yakutia was chosen. As the model was parameterised for northern distribution areas of the study species L. *gmelinii*, the model had to be newly parameterised to be applicable in the south.

We incorporated an abstracted drought resistance trait with a value from 0 to 100, with 100 representing drought immunity. This trait is negatively correlated with the growth rate of trees as they need to invest in this adaptation. In the model the trait represents an unspecific resistivity to drought/fire which has, in reality, multiple sources. That larch can adapt to drought was shown in studies for *Larix decidua* (Plesa et al., 2019; George et al., 2019). New parameterisation was needed after introducing the drought resistance trait, as the drought calculation present no longer gave reasonable results in the test described below. In LAVESI, drought is calculated by using the monthly temperature and precipitation data and calculating a general drought mortality for each year. A decreasing function with a limit of zero based on tree height is then used to calculate drought mortality of individual trees for a given year. This drought mortality is then included in the calculations determining if a tree dies that year for any reason, thus larger numbers lead to more tree death (Kruse et al., 2016).

To parameterise the model to the southern species distribution we created four transects (Appendix Fig. 6) across the species line (4 plots above and below the species line per transect), using both species distribution maps (Mamet et al. 2019) and satellite images (source Google Earth) to determine where the species can be found. This enables us to find a parameterisation for the drought components by using a pattern-orientated modelling approach (Gallagher et al., 2021). These plots are simulated and forced with CRU TS weather data of random years (1901–2018) on an area of 200 m x 200 m and inheritance data resulting in either a population or not (Appedix Fig. 7). The simulations that predict presence/absence are achieved by altering the drought in two separate places. To determine the values, a range of values was tested and the best fit calculated. The drought strength present in an area is first calculated based on temperature and precipitation. If the evaporation rate exceeds input by precipitation, the drought strength is calculated based on the active air temperature once a certain value is reached. This overall drought strength is, for this version after parametrisation (Appendix Fig. 8), increased by applying the formula (drought/0.3863)². The second change is in the formula estimating the individual drought strength applied to a tree. In the previous version of the model, the drought strength decreases as the tree grows taller, via multiplication with the formula $\sqrt{1.0/heightoftree}$. In this version, a value of 0.5 is added after this step to increase the amount of drought stress applied to the trees and ensure even very tall trees are still meaningfully affected, whereas previously large trees became effectively immune to drought. After this calculation, the drought resistance factor is applied to get the final drought mortality.

### 4.2 Simulations for the drought

When running simulations for a region, first the current drought resistance of the population is determined from simulations forced using randomised TraCe (He 2011) data from 1 to 1900 CE for a given location for a period of 10 000 years and allowing the trees to adapt to the conditions. This assumes that the trees would be adapted to the conditions that were present before the 20th century. Over time, the yearly average trait value for drought resistance of new trees oscillates around a value that is assumed to be the optimal trait value for the location. The average is calculated from the values from simulation years 4000–10 000. This optimum is then used as the value for uniform tests and as the centre point for the distribution used by both variation and (for initial seeds) the inheritance simulations.

**Fig. 2:**
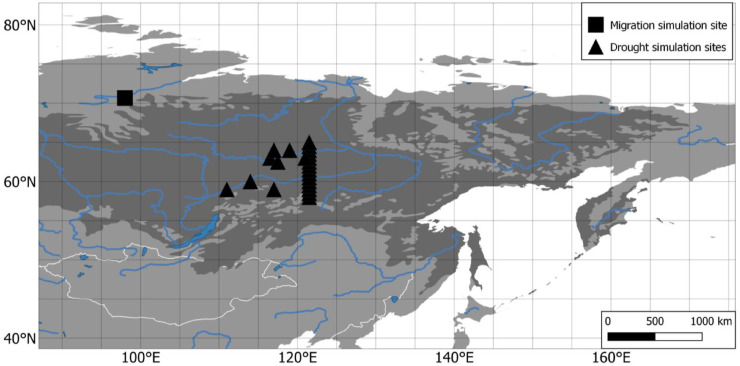
Overview map with markers of simulation areas for treeline migration in the north and drought impact in the south of the boreal forest. The dark grey area shows the boreal forest area. The white lines show borders and the blue lines rivers and lakes.

The simulations are performed using a transect representing a slice of an area expected to be affected by droughts in the future, based on the calculated drought strengths for the area. To simulate a wide range of conditions, the transect is split into individual simulations, as the length of the simulated transect is too limited to cover the desired range of drought conditions. To cover an even wider range of conditions, more plots randomly distributed in the surrounding area of the transect are included (Fig. 2). For each grid-cell of weather data a simulation is performed on an area of 400 m x 400 m. For the first 1000 years, simulations are forced with random years selected from weather data from CRU TS covering 1901–2018. The first 1000 years are both to simulate the current conditions of the populations and as the spin up-phase to allow the forests to establish from seeds introduced over the entire area. The subsequent 2000 years use the same data but with the temperature and precipitation raised to the level predicted by the CMIP5 (Giorgetta et al., 2012) data using the RCP 4.5 scenario for 2260–2300 CE, as it covers both strong and weak drought over the area. The scenarios RCP 2.6 and RCP 8.5 only showed the corresponding extreme results. Each of the simulation scenarios for every plot was repeated ten times to counteract variation.

For comparison of the drought die-back rates revealed by the simulation setup, a quasi-binomial model was established using the percentage of the average number trees that are over 1.3 m tall in the last 200 years of the simulation and comparing it to the average of the last 200 years of the spin-up phase, that is, under the current climate conditions. The drought strength is the average value calculated by the model for the simulation years. These models are created for all three scenarios.

The spatial upscaling of the simulation results was performed by calculating the drought value for every cell with a size of 0.5° x 0.5° using the same formula as in LAVESI and survival curves, from which the expected population change is calculated. When a population size falls below 5%, the population is considered extinct. The comparative results are shown limited to the current area of boreal forest for the region (Fig. 2) (Olson et al., 2001).

## 5 Results

### 5.1 Impact of trait value variation and inheritance on larch forest dynamics in the focus regions

**Fig. 3:**
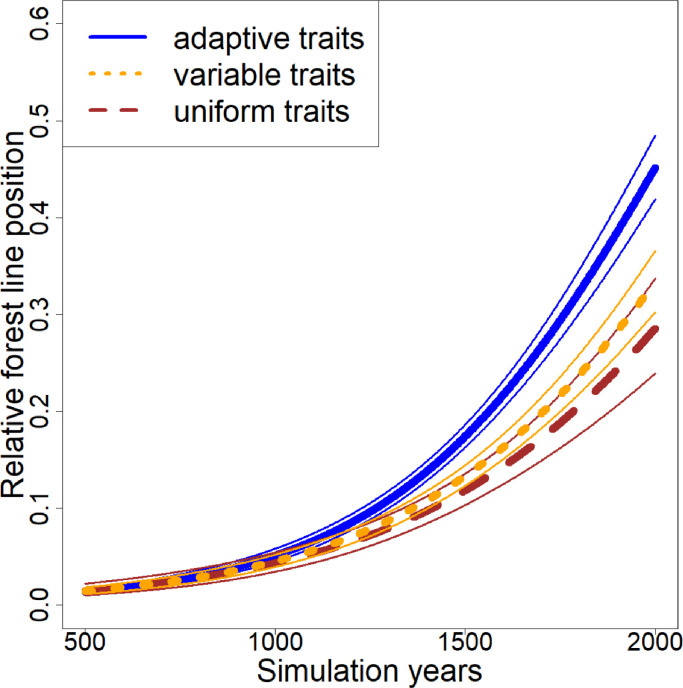
Relative position of the forest line over time using three trait scenarios. The quasi-binomial regression lines of the three main scenarios are shown over the simulation length starting from the end of the spin-up phase. The larger lines given in the legend show the regression, while the thinner unbroken lines of matching colours show the standard error. A relative position of 0.1 equals 1 km of the simulation area.

The three trait scenarios – uniform, random variation, and inherited traits – lead to a difference in the percentage area the forest line reaches, which increases over time (Fig. 3). A value of 0.5 indicates that the tree line has reached up to half the length of the transect. Initially, the area covered by forest is the same in all scenarios and continues to slowly increase after spin-up of 500 years but they diverge around simulation year 900. In the later stage, the migration rate increases further and both the randomly variable traits and inheritable adaptive traits scenarios increase faster than the uniform traits scenario. Adaptive traits have the fastest migration rates and farthest forest cover extent of the simulation transect. The average seed weight decreases under the adaptation variant over time.

**Fig. 4:**
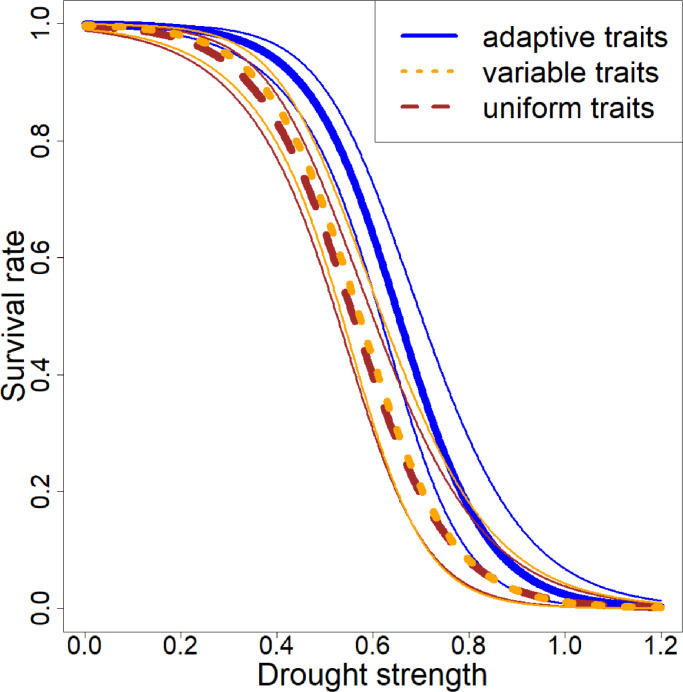
Comparison of the survival rate of populations (calculated from population sizes before temperature increase and at the end of the simulation) in three trait scenarios for different levels of drought severity experienced under the climate of the RCP 4.5 for the period 2260–2300. The larger lines given in the legend show the regression, while the thinner unbroken lines of matching colours show the standard error.

Survival under different drought strengths, based on different simulation plots using the RCP 4.5 scenario weather data, decreases with increasing drought (Fig. 4). Earlier offset of drought affecting the population sizes can be seen at lower drought strength in the uniform and randomly variable drought resistance scenarios, while populations with adaptive trait values are more resilient. In those adaptive populations, the average drought resistance increases after the drought increase. Once the drought reaches a value of 1.2, all populations are expected to have died out with some already having died at a value of 1.

### 5.2 Siberian-wide impact of adaptation on future larch forests

**Fig. 5:**
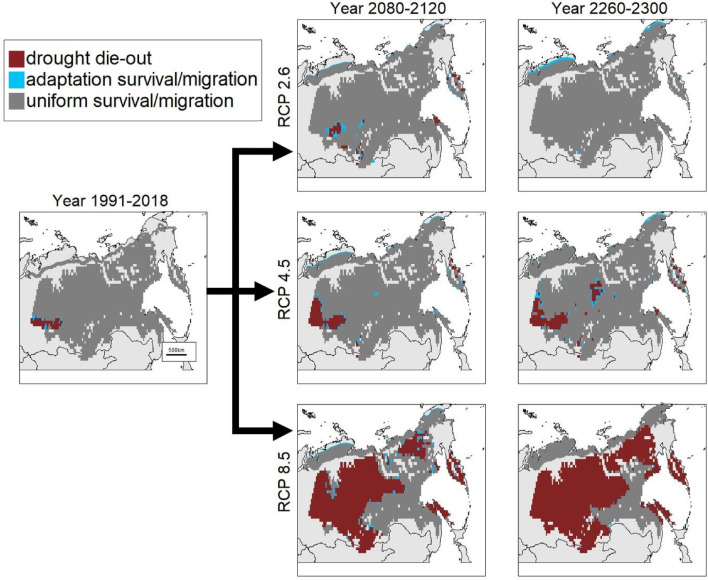
Overview of forest response over three warming scenarios – RCP 2.6 (top row), 4.5 (middle row), and 8.5 (bottom row) for selected time intervals. The Albers equal area projection was used for these maps. Areas not marked with a colour shown in the legend were outside the scope of analysis.

The results extrapolated from the two results above onto the larger area show that the harsher the RCP scenario used for predictions is, the faster the migration is and the stronger the drought die-out (Fig. 5, Table 1). Predictions further into the future generally lead to larger changes, but to smaller effects from adaptation through migrations as the species is expected to have reached the coastline in most places. The exception to this is RCP 2.6 in which there is a reduction of drought severity and the adaptive migration has the largest impact.

**Table 1:** Area of forest response for the different predictions for both migration (left) and survival (right). The percentages are the respective cover compared to migration/survival.

	Migration[km²]	Adaption enabled migration[km²]	Survival[km²]	Adaption enabled survival[km²]
present	455,218	0 (0%)	6,742,889 (96.8%)	40,104 (0.6%)
RCP 2.6				
2100	917,305	43,323 (4.5%)	6,660,007 (95.6%)	106,945 (1.5%)
2300	1,055,750	108,617 (9.3%)	6,956,780 (99.9%)	5347 (0.1%)
RCP 4.5				
2100	994,612	44,553 (4.3%)	6,427,401 (92.3%)	77,535 (1.1%)
2300	1,247,443	35,778 (2.8%)	6,200,143 (89.0%)	131,008 (1.9%)
RCP 8.5				
2100	1,051,372	42,916 (3.9%)	2,759,184 (39.6%)	157,744 (2.3%)
2300	1,314,755	0 (0%)	1,470,495 (21.1%)	18,715 (0.3%)

The results obtained using the climate data from the RCP 4.5 scenario and the time frame of 2260–2300 shows an interestingly varied response. There are many areas that are estimated to have surviving populations although the estimations for many of the locations lead to lower population sizes when using the uniform traits compared to the adaptation traits. Up to 11% of the area is estimated to have populations go extinct when using only uniform traits for simulations, but 17% of this area (2% of the total population area) is expected to survive when factoring adaptation into the extrapolation. For migration, having adaptive traits is expected to extend the range in areas where the treeline has not yet reached the ocean by an area of 35 778 km² (Table 1).

## 6 Discussion

### 6.1 Impacts of trait value variation and inheritance on larch forest dynamics

Our results suggest that the migration rate of forests into northern tundra areas is faster for scenarios with seed-weight variation. This effect is even stronger in the adaptation scenario since the average trait value of the populations can change due to heritability of the seed characteristics, as was found by Barchenkov (2011). The offspring of a tree with light seed has a greater chance of migrating farther north and also has a greater chance of producing light seeds themselves. This leads first to a decrease in seed weight as those seeds are spread farther and cover larger areas and in consequence a faster migration can be observed as the overall lighter seeds are spreading farther. This continues until the simulation area is covered. Thus, inheritance combines the average seed weight, variation, and local fine-scale adaptation and it can ultimately influence the simulated migration rate. The observed migration rate (McDonald et al., 2008; Kharuk et al., 2018) already includes such variation.

Warmer temperatures increase the migration rate and lead to the populations reaching the coastline faster. This is due to warmer climate leading to faster tree growth and thus allowing the trees to reach their seed-producing stage earlier and to be more prolific (Kruse et al., 2019; Kruse and Herzschuh 2022). Therefore, an increase in temperature not only leads to a larger inhabitable area, but also to faster migration into an area that is already habitable but not yet colonised (McDonald et al., 2008). The simulations can be influenced by long-distance dispersal events (Fayard et al., 2009), which could lead to some heavier seeds randomly being transported a long way. Therefore, adaptation can sometimes lead to slower expansion if a population initially has heavier seeds, although the general trend is towards faster migrations as long-distance dispersal events are more likely with lighter seeds in our model. Previous studies have estimated that the time lag caused by the slow migration of trees could lead to grassland forming in the warming northern areas (Chapin and Starfield 1997). Our study shows that this might not happen because of the faster migration of the trees when considering variation and adaptation.

In areas that experience a strong drought, the population size is reduced due to increased tree mortality. In the inherited adaptation and random variation scenarios there is a range of different drought adaptations leading to the possibility of pre-adapted trees being present. Initially, in both scenarios some trees survive. However, in the adaptation scenario the offspring are more likely to be drought adapted as well, whereas the offspring in the variation scenario have the same chance of being adapted as their parents and with increased change in drought strength the likelihood of this being a useful adaptation becomes smaller. Therefore, the number of viable offspring is much smaller under the variation scenario and the population cannot recover their numbers easily and is more likely to die out due to random events. The model behaves reasonably with respect to droughts and the simulation results match observations that show smaller populations of Siberian larches are more affected by drought (Khansoritoreh et al., 2017). Populations that are affected by disturbances have fewer individuals and denser tree stands lead to shallower permafrost thaw (Kropp et al., 2019), thus impacting the landscape and carbon stored. Furthermore, droughts weaken pine populations, which are exacerbated by secondary effects, such as insects or fungi, leading to a higher mortality (Kharuk et al., 2013), with warmer temperature also making natural disturbances more likely in boreal forests (Seidl et al., 2020). Therefore, simulations that result in a local population barely surviving could be interpreted as a threat of extinction which is likely to cause reduced forest survival and probably prevent recolonisation under such conditions.

This study finds a larger increase in temperature is needed to lead to drought-related die-out compared to a previous study (Brazhnik and Shugart 2015). This could be due to the different study areas and their inclusion of terrain but might also stem from our assumption that the trees are adapted to the conditions from before the 20th century. The strong difference in forest dieback between the three scenarios underlines the importance of an accurate prediction of the climate. Currently there is some evidence for RCP 8.5 to be the closest (Schwalm et al., 2020), which would mean that greater areas of the current larch population could be threatened.

The inclusion of variation and adaptation of key traits in an individual-based model affects modelled population dynamics. As in nature, variation of traits in our model allows a local population to include a range of drought resistances and survive smaller droughts (Ovenden et al., 2021) and inheritance allows it to adapt to the prevailing conditions (Savolainen et al., 2007). Thus, the model only needs to have a small range of variation around the optimum along with the possibility for a population to be optimised for past conditions in a fast-changing environment (Svenning and Sandel 2013). When including traits in a model using adaptation it is vital to ensure the traits represented in the model are inheritable as some traits can be almost exclusively influenced by the environment (Geber and Griffen 2003) and the degree of this phenotypic plasticity can be limited by other factors (Valladares et al., 2007). Differences in both seed weight (Wieczorek et al., 2017; Barchenkov 2011) and drought resistance (Kagawa et al., 2006) have been found between populations of *Larix gmelinii.* In general, *Larix gmelinii* does have genetic variation between and within populations (Larionova et al., 2004; Zhang et al., 2013).

### 6.2 Siberian-wide impact of adaptation on future larch forests

We could show that the impact of warming is meliorated with the adaptation of key traits. While more inhabitable areas in the north might open up for tree growth the migration rate might not be able to keep up with the changing possible range (Kruse et al., 2016). The response in the south, however, will be faster once the trees start dying from drought but could be delayed with a weaker decline if temperature rises only gradually and trees are able to adapt. At some point a breakpoint will be reached and the population disappears. Where this breakpoint lies depends on the rate of adaptation in the tree populations. Simulations ignoring adaptation are going to place the threshold at a lower level than is realistic. As populations cannot migrate further north indefinitely as the coastline prevents expansion north (Mamet et al., 2019), drought triggered die-out could affect large parts of the population if temperatures increase to the level of RCP 8.5, as predicted by Schwalm et al. (2020), hence migration north cannot offset the dieback south indefinitely.

Our simulations show the potential future development *Larix gmelinii*. While this larch species and other similar larch species are dominant over large areas, especially at the northern margin (Abaimov et al., 1998), in the west and south mixed forests with evergreen taxa prevail. This means that the predictions can only be taken as predictions for the survival of larch species in northern areas and therefore the overall forest cover might react differently. While it is known that other species of the biome are experiencing droughts as well (Kharuk et al., 2017), both Shuman et al. (2011) and Tchebakova et al. (2016) predict a change in forest type for their study region that depends upon the climate scenario used. Mixed forests could also increase the likelihood of larches dying out as the current simulations only consider pure larch forests, and as discussed previously, smaller populations that are more isolated are more likely to die out and the effect of future climate change might be more adverse on larches in mixed forest. Mixed forest can also directly increase the drought that larch experiences because some species cause the soils to dry out more (Bonan 2008). This could lead to stronger effects on larches in mixed forest than predicted.

Our simulation study shows that the majority of tree-cover change depends largely on the actual change in climate. The stronger the climate changes, the more drastic the effects can be expected. Although initial increases in temperature can result in better tree growth (Zhang et al., 2019), further increases lead to drought (Zhirnova et al., 2020). In addition, topography can play a role in migration and larch abundance, as passing a mountain range is more challenging than crossing flat terrain (Macias-Fauria and Johnson 2013; Sato and Kobayashi 2017). Our simulations and the resulting extrapolations do not take topography into account explicitly, yet the underlying climate data reflect the broader changes.

### 6.3 Modelling trait adaptation to climate change

Random variation and inheritable adaptation of traits allows for a range of trait values to be present within the simulated population when change occurs, be it to allow for new growth or an adverse effect on plant health. This does come with two related issues: the first being lower population numbers, as only a few trees are well suited to any one circumstance, and the second being that all trait variations are always available. This can lead to populations surviving even if the changes are very drastic and happen quickly. Both these shortcomings are addressed by the introduction of inheritable adaptive traits. Hence, our model allows for small-scale local adaptation and for variation in traits to coexist within the same area, as long as the advantages are not too unequal. While our study already discusses changes to a large ecosystem that could impact global systems (Schaphoff et al. 2016; Bonan 2008), the implemented functions combined with dispersal and pollination (Kruse et al., 2018) could be applied to other models and contribute to research of further global problems.

For this research, we limited the scope to one adaptable trait per region (seed weight in the north and drought tolerance in the south) to reduce variation between the simulations and thus allow for a clearer differentiation of the effects. While it is possible to include more larch species and, with recent developments, many boreal tree species (Kruse et al., 2022), this study uses the species the model as was originally parameterised (Kruse et al., 2016) since L. *gmelinii* is the dominant species over vast areas of eastern Siberia (Abaimov et al., 1998).

## 7 Conclusions

The inclusion of random variation and inheritable adaptive traits has the potential to change the results of predictions to an extent that warrants future adoption of the process in studies allowing more dynamic responses of trees to be modelled. Simulations over long timescales or of taxa with widely varying traits are greatly enhanced by these new inclusions, because species are able to adapt to changed conditions but also appear maladapted if changes are happening too fast. When only variation is used, maladaptation cannot be simulated, especially at the regional scale. The limiting factor for inclusion is the increased amount of computational resources required to perform the additional calculations to determine the pollen source and the new trait values. Thus, small-scale simulations with either few generations or a small area may not gain sufficient benefit to warrant the new inclusion of adaptation.

From our results, we could infer that while larch forests are able to spread farther north with increasing temperature, this spread is ultimately limited and cannot, under the RCP 8.5 scenario, offset the potential loss of large forest areas due to drought. The rate at which this predicted change happens is not only affected by climate but also by the choice of trait parameterisation in the simulations.

With the inheritance of traits, new possibilities for simulations arise. Changes over long time-scales or heterogeneous landscapes can be simulated with the trees adapting to local or temporal conditions. These simulations can later be expanded by introducing trees from other populations or more drought-adapted species to analyse how they affect the results, with the output informing forestry management.

## Data archiving statement

Before publication the authors will upload the data created during simulations and the code used for the model to Zenodo.

## CRediT authorship contribution statement

**Josias Gloy:** Methodology, Software, Formal analysis, Investigation, Visualization, Writing – original draft, Data curation. **Ulrike Herzschuh:** Conceptualization, Funding acquisition, Writing – review & editing, Resources. **Stefan Kruse:** Conceptualization, Supervision, Writing – review & editing, Resources.

## Declaration of Competing Interest

The authors declare that they have no known competing financial interests or personal relationships that could have appeared to influence the work reported in this paper.

## Data Availability

The code and simulation data will be made available. The weather data used is available from the cited sources.

## Appendix Supplementary materials

Image, application 1

## Footnotes

Supplementary material associated with this article can be found, in the online version, at doi:10.1016/j.ecolmodel.2023.110278.
