# Supplementary material for: Evolutionary adaptation of trees and modelled future larch forest extent in Siberia
Source: Ecol Modell. 2023 Apr;478:110278. doi: 10.1016/j.ecolmodel.2023.110278 (PMC9972785; doi:10.1016/j.ecolmodel.2023.110278)
Supplement: Supplementary file 1 [file mmc1.docx]

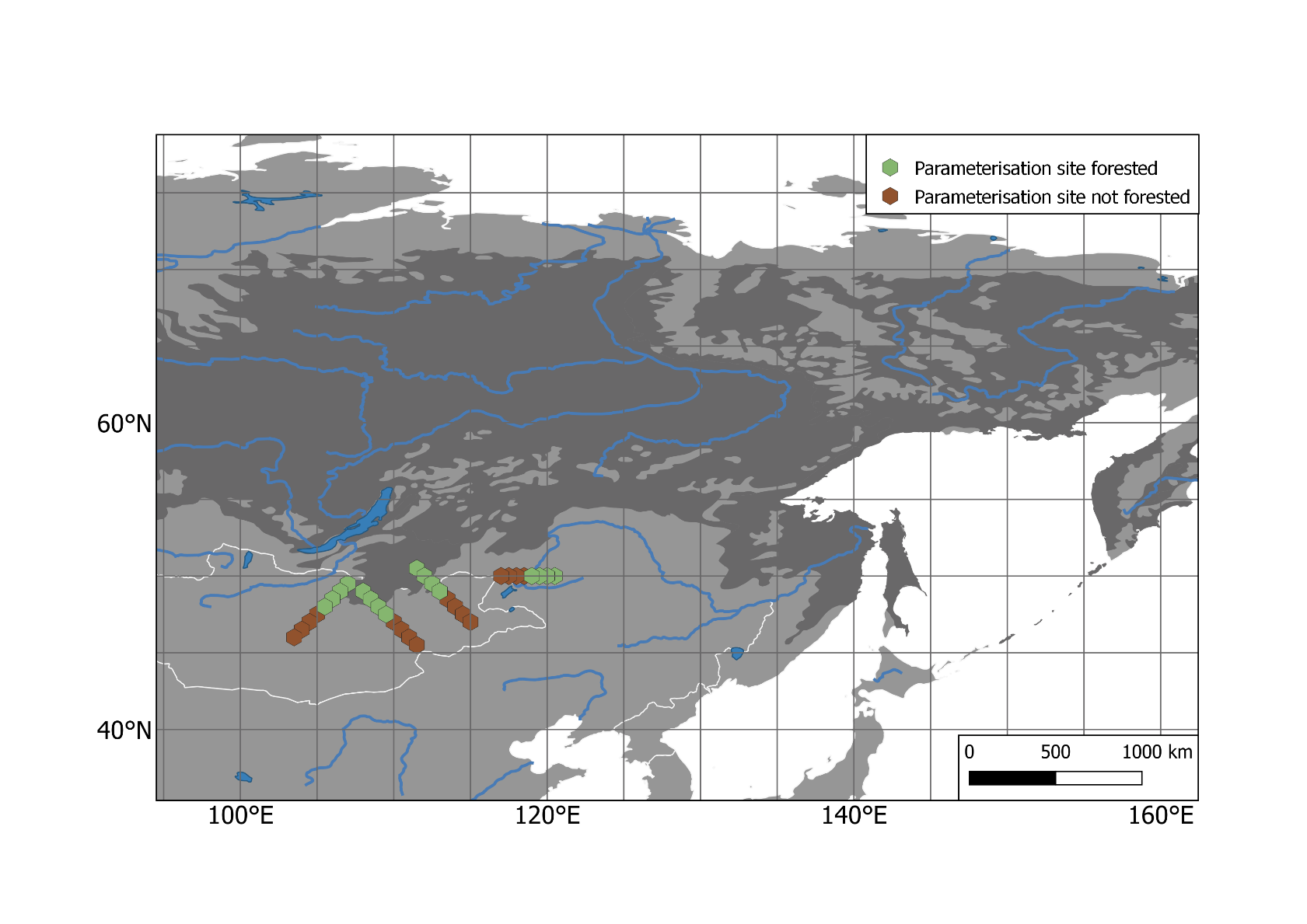


Figure 6 Map showing the four transects used for the parameterisation of the drought strength. The green symbols are forested sites and the brown symbols are not forested.


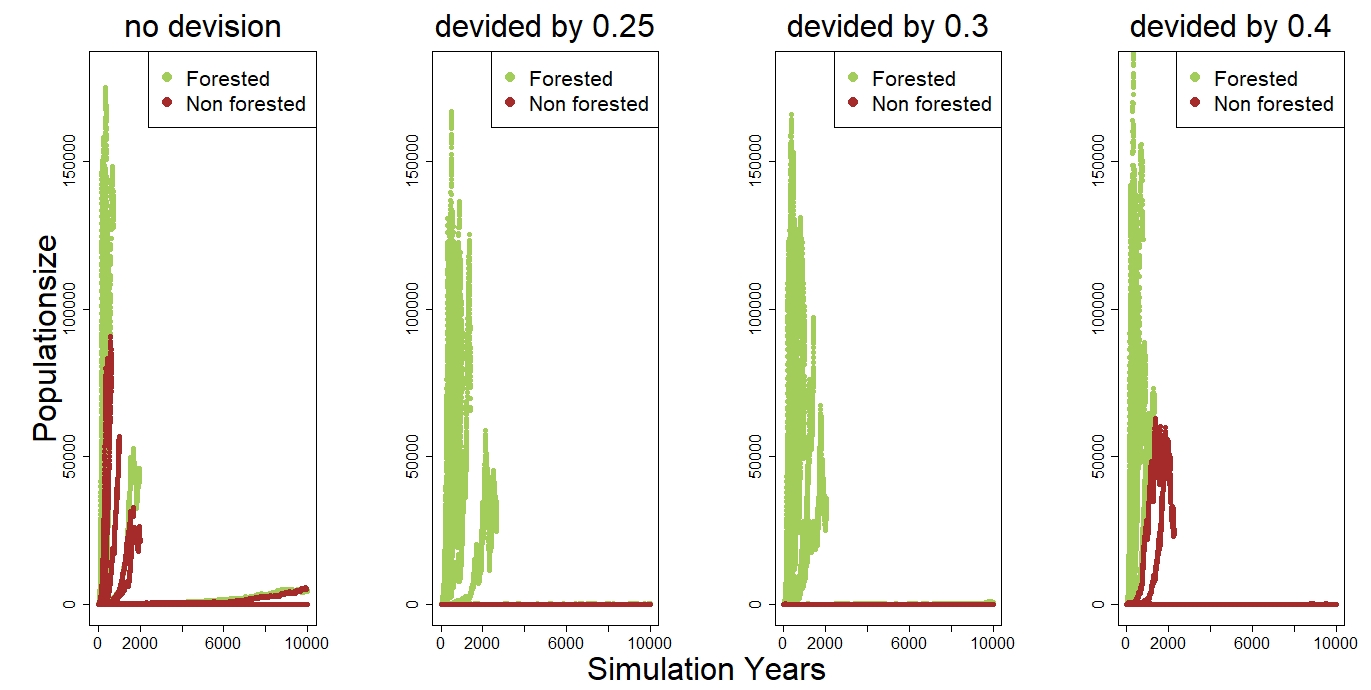


Figure 7 presence/absence data for the sites of Figure 6.


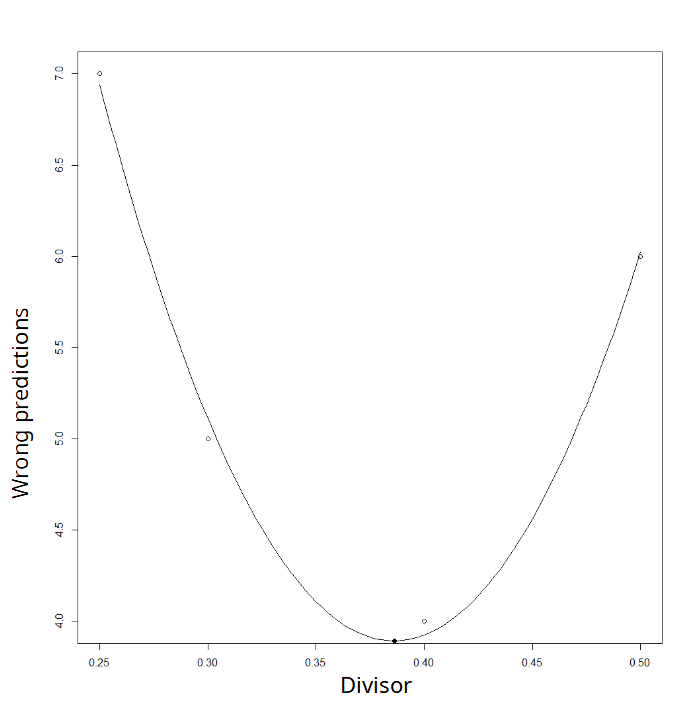


Figure 8 Regression based on Figure 7 with the Divisor and the wrong predictions made by the model
